# Multimodal diagnosis model of Alzheimer’s disease based on improved Transformer

**DOI:** 10.1186/s12938-024-01204-4

**Published:** 2024-01-19

**Authors:** Yan Tang, Xing Xiong, Gan Tong, Yuan Yang, Hao Zhang

**Affiliations:** 1 School of Electronic Information, Central South University, Changsha, 410008 Hunan People’s Republic of China; 2 School of Computer Science and Engineering, Central South University, Changsha, 410008 Hunan People’s Republic of China; 3 Guangxi Key Lab of Multi-source Information Mining & Security, Guangxi Normal University, Guilin, 541004 Guangxi People’s Republic of China; 4 Department of Bioengineering, University of Illinois Urbana-Champaign, Grainger College of Engineering, Urbana, IL USA

**Keywords:** Alzheimer’s disease, Deep learning, Multimodal medical images, 3DCNN, Transformer, Visualization

## Abstract

**Purpose:**

Recent technological advancements in data acquisition tools allowed neuroscientists to acquire different modality data to diagnosis Alzheimer’s disease (AD). However, how to fuse these enormous amount different modality data to improve recognizing rate and find significance brain regions is still challenging.

**Methods:**

The algorithm used multimodal medical images [structural magnetic resonance imaging (sMRI) and positron emission tomography (PET)] as experimental data. Deep feature representations of sMRI and PET images are extracted by 3D convolution neural network (3DCNN). An improved Transformer is then used to progressively learn global correlation information among features. Finally, the information from different modalities is fused for identification. A model-based visualization method is used to explain the decisions of the model and identify brain regions related to AD.

**Results:**

The model attained a noteworthy classification accuracy of 98.1% for Alzheimer’s disease (AD) using the Alzheimer’s Disease Neuroimaging Initiative (ADNI) dataset. Upon examining the visualization results, distinct brain regions associated with AD diagnosis were observed across different image modalities. Notably, the left parahippocampal region emerged consistently as a prominent and significant brain area.

**Conclusions:**

A large number of comparative experiments have been carried out for the model, and the experimental results verify the reliability of the model. In addition, the model adopts a visualization analysis method based on the characteristics of the model, which improves the interpretability of the model. Some disease-related brain regions were found in the visualization results, which provides reliable information for AD clinical research.

## Background

Alzheimer’s disease (AD) is a major neurocognitive impairment, which is the most common cause of dementia in people over the age of 65 [1]. It is usually manifested in the changes in memory, abstract thinking, judgment, behavior, and emotion, and finally interferes with the physical control of the body [2]. However, the diagnosis of AD often requires physicians to use various clinical methods including medical history, mental status tests, physical and neurological exams, diagnostic tests, and brain imaging [3]. Therefore, a computer-aided AD diagnosis is in urgent need of objective and efficient methods.

Medical imaging technology is a powerful tool to identify the progression of brain diseases. More specifically, magnetic resonance imaging (MRI) and positron emission tomography (PET) can assist in diagnosing the disease and monitoring its progress [4]. Structural MRI (sMRI) can well-quantify brain tissue atrophy in patients with AD [5]. KLöppel et al. [6] generated the gray matter density map of the brain using the sMRI images of subjects and realized the identification of AD using the support vector machine (SVM). The PET can monitor the changes in glucose metabolism in the human body [7]. Wen et al. [8] extracted PET image features and identified AD from healthy controls by logistic regression. For the features of a single modality, the observed feature information usually only is provided from a certain perspective. The feature information of multiple modalities can realize a more comprehensive study of human brain. Therefore, developing AD diagnosis models based on multimodal medical images has become a new trend. A few recent studies show that multimodal brain imaging data results have better performance than single-modal data [9–11].

Some AD-related networks have been discovered and new insights have been provided for the pathological mechanisms of AD with seed-based methods such as hippocampus volume, regional cortical thickness, and temporal lobe. For example, Ardekani et al. proposed a method of segmenting the hippocampal region to identify AD [12]. Williamson et al. introduced a connectivity analysis to identify sex-specific AD biomarkers based on hippocampal–cortical functional connectivity [13]. However, mounting evidence indicates that neurodegenerative processes, even if they are highly localized, are associated with disease-specific alterations across the whole brain [14, 15]. Thus, the abnormal patterns of AD across the whole-brain scale have yet to be investigated.

Recently, the deep learning approach has attracted a lot of attention to exploring new imaging biomarkers for AD diagnosis and prediction, which requires no prior knowledge to extract biologically meaningful information from subtle changes. Zhang et al. [16] proposed a deep learning framework based on gray matter slices from sMRI. This framework combines slices with attention mechanisms and achieves AD classification through residual networks. However, this slice-based approach leads to the loss of spatial information in 3D brain images, thereby affecting the classification performance. Therefore, Feng et al. [17] proposed to use a 3DCNN to extract features from MRI and PET images. They cascade these features and then use a stacked bidirectional recurrent neural network (SBi-RNN) to obtain further semantic information for identification. However, SBi-RNN has the problem of gradient explosion or gradient disappearance. To address this challenge, Feng et al. [18] proposed to use a 3DCNN to extract features from MRI and PET images. They use fully stacked bidirectional long short-term memory (FSBi-LSTM) to extract all the spatial information from the feature maps and further improve the performance. However, there are direct or indirect connections between different feature maps, and these global correlations are ignored by the above research.

The transformer model was first proposed by Vaswani et al. [19]. It has a powerful capability of global information integration. The self-attention mechanism in it can quickly obtain the global correlation between input features without stacking many layers like CNN, and these computations are all parallel [19]. Thus, it can effectively capture the non-local relationships among all input feature maps. This mechanism also makes the model more interpretable. Vision Transformer (ViT) is a pioneering work of transformer in the field of computer vision, it and its variants have shown excellent performance in various image-related tasks [20–22]. However, the original ViT only deals with 2-D images, and the input is the sequence of linear embeddings of image patches [20]. In our scenario, the input is 3-D medical images, and directly patching would destructing the connection among brain areas. Hence, we employ 3DCNN to extract features that serve as input to the Transformer for AD diagnosis. However, the features extracted by 3DCNN in the initial stage of model training are not representative, and learning their global information is meaningless. Therefore, we optimize the transformer by gradually introducing a self-attention mechanism to help model training focus more on feature extraction in the initial stage.

In summary, we proposed a network model based on 3DCNN and Transformer for AD diagnosis. In specific, an improved Transformer is used to learn the correlation information among features, which are extracted from medical images by 3DCNN. The information from different modalities is fused and identified through the fully connected layer. We conducted extensive experiments on the publicly available ADNI dataset, and the model demonstrated excellent performance. In addition, we performed interpretability analysis of the model's decisions using visualization methods based on its characteristics and identified several brain regions associated with AD.

## Results

### Performance of the proposed method

We obtained the identification performance with the ACC of 98.10% (precision 99.09%, SPE 96.75%, SEN 96.75%, F1 97.81%, AUC 98.35%) in AD diagnosis (see Table 1).

**Table 1:** Network performance in different settings (mean ± standard deviation, %)

Methods	ACC	PRE	SPE	SEN	F1S	AUC
Proposed method	98.10 ± 2.46	99.09 ± 2.87	96.75 ± 5.28	95.82 ± 5.03	97.81 ± 2.87	98.35 ± 2.14
Only sMRI	91.91 ± 5.96	91.05 ± 10.49	92.72 ± 8.67	90.91 ± 9.41	90.31 ± 6.16	91.33 ± 5.99
Only PET	87.14 ± 7.12	85.4 ± 10.31	88.62 ± 8.59	85.05 ± 15.61	83.99 ± 9.80	87.43 ± 6.61
Typical Transformer	94.32 ± 4.01	93.57 ± 7.76	93.33 ± 6.34	92.25 ± 7.36	93.40 ± 4.25	95.05 ± 3.11
Without Transformer	92.86 ± 7.12	90.60 ± 10.31	92.20 ± 8.59	92.03 ± 15.61	90.78 ± 9.80	93.11 ± 6.62
Without 3DCNN	90.01 ± 3.52	83.44 ± 8.77	89.46 ± 5.05	93.02 ± 7.59	87.45 ± 4.22	90.06 ± 3.48

*ACC* accuracy, *PRE* precision, *SPE* specificity, *SEN* recall/sensitivity, *F1S* F1 score

**Fig. 1:**
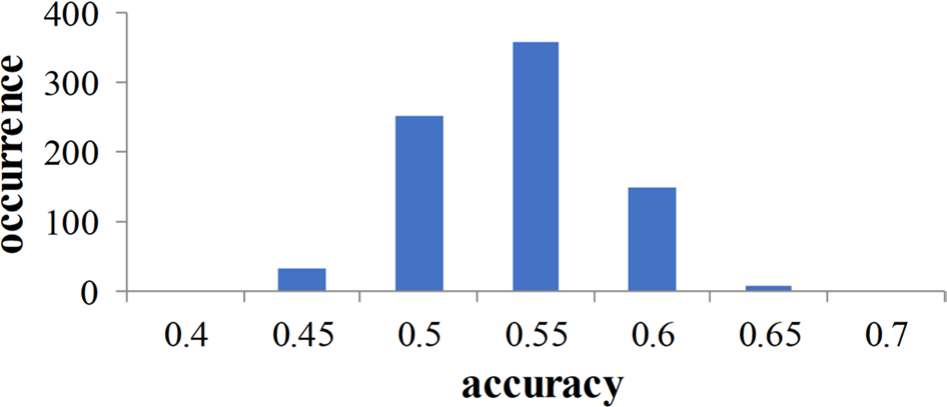
Permutation distribution of the estimate

First, we employed permutation tests to assess the statistical significance of the tenfold cross-validation results. For each tenfold cross-validation, the identification labels of the training data were randomly permuted 1000 times. The null hypothesis is that identifier cannot learn to predict labels based on the given training set. Experiments show that for each training, usually the training set converges or even overfits, while the validation set does not converge. With accuracy as the statistic, the result (see Fig. 1) of permutation distribution of the estimate revealed that this identifier learned the relationship between the data and the labels with a risk of being wrong with a probability of lower than 0.01.

Then, we conducted a series of comparative experiments as follows. To demonstrate the superiority of multi-modal fusion, we implemented two single-mode variants of our method (only sMRI and only PET). Thus, the model was unchanged except for the multimodal fusion part. We trained the model with single-mode data (sMRI or PET) and take the results of a tenfold cross-validation. The results are also shown in Table 2, from which we can find that the multimodal fusion method significantly improved the performance. Compared with the sMRI only variant, the accuracy was improved by 6.19%, while the improvement over the PET only variant is 10.96%.

**Table 2:** Cluster distribution statistics of sMRI 142nd feature deconvolution map in brain regions (5 clusters)

Region	MNI coordinates			Peak intensity	Voxels
	*x*	*y*	*z*		
Right fusiform	34	− 4	− 43	2.89	72
Right cerebellum posterior lobe	39	− 78	− 35	2.25	71
Left middle temporal gyrus	− 51	−	− 22	2.31	71
Left parahippocampal	− 22	− 8	− 22	2.14	69
Right inferior frontal gyrus	32	36	− 10	2.24	154

**Fig. 2:**
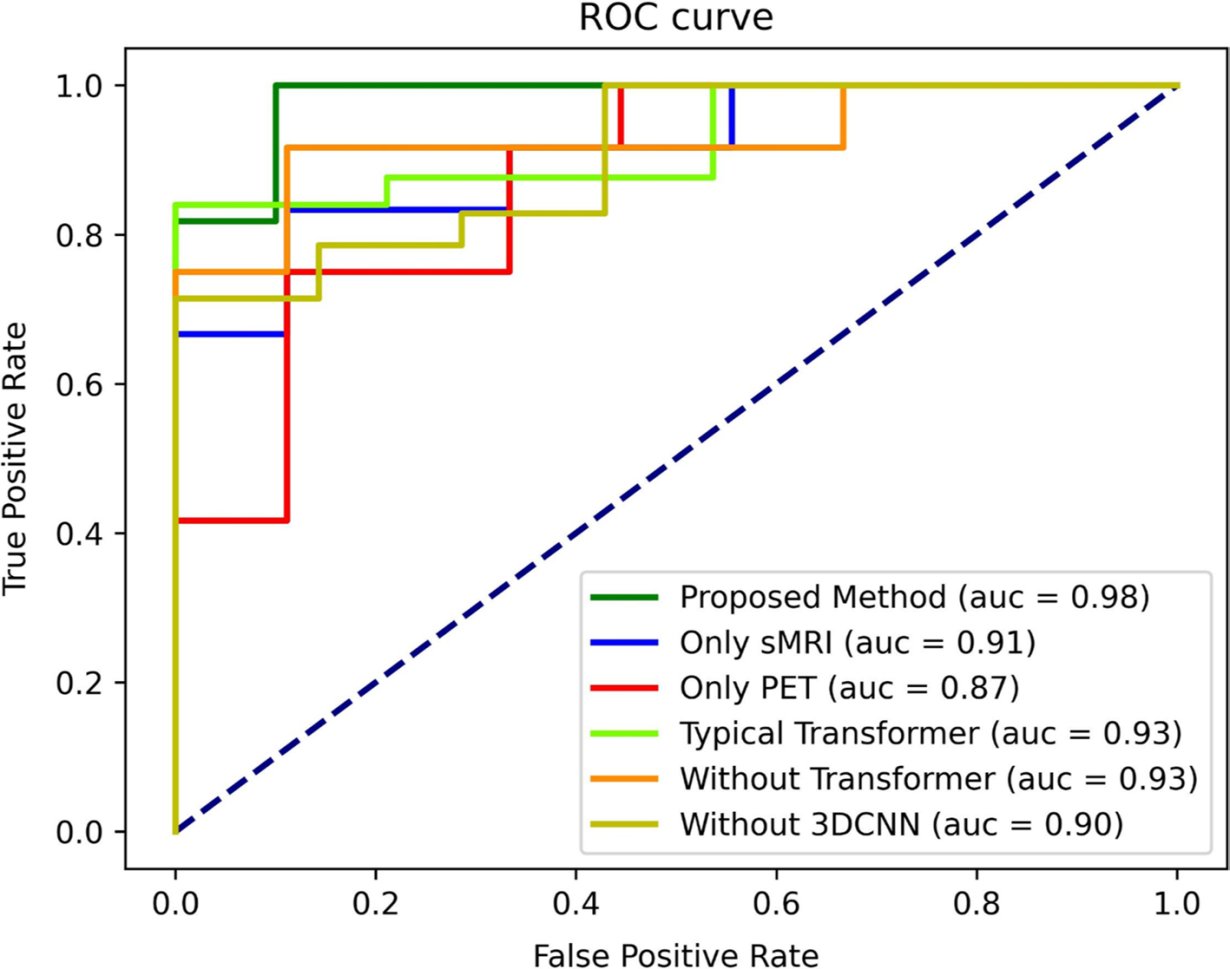
ROC curves of different methods

In addition, compared with the classical Transformer, our improved solution also shows better performance (3.78% higher in accuracy). When the model only included the 3DCNN by excluding the Transformer part, the accuracy of identification is 5.24% lower. We also tried to remove the 3DCNN part from the model. We followed the approach in ViT [20]: split the 3D image into patches and added positional encoding before inputting them into the Transformer. The results show that the identification accuracy of the model without 3DCNN is 90.01%, which verified the importance of feature extraction using 3DCNN. The ROC curves of different methods are shown in Fig. 2.

### Visualization results

**Fig. 3:**
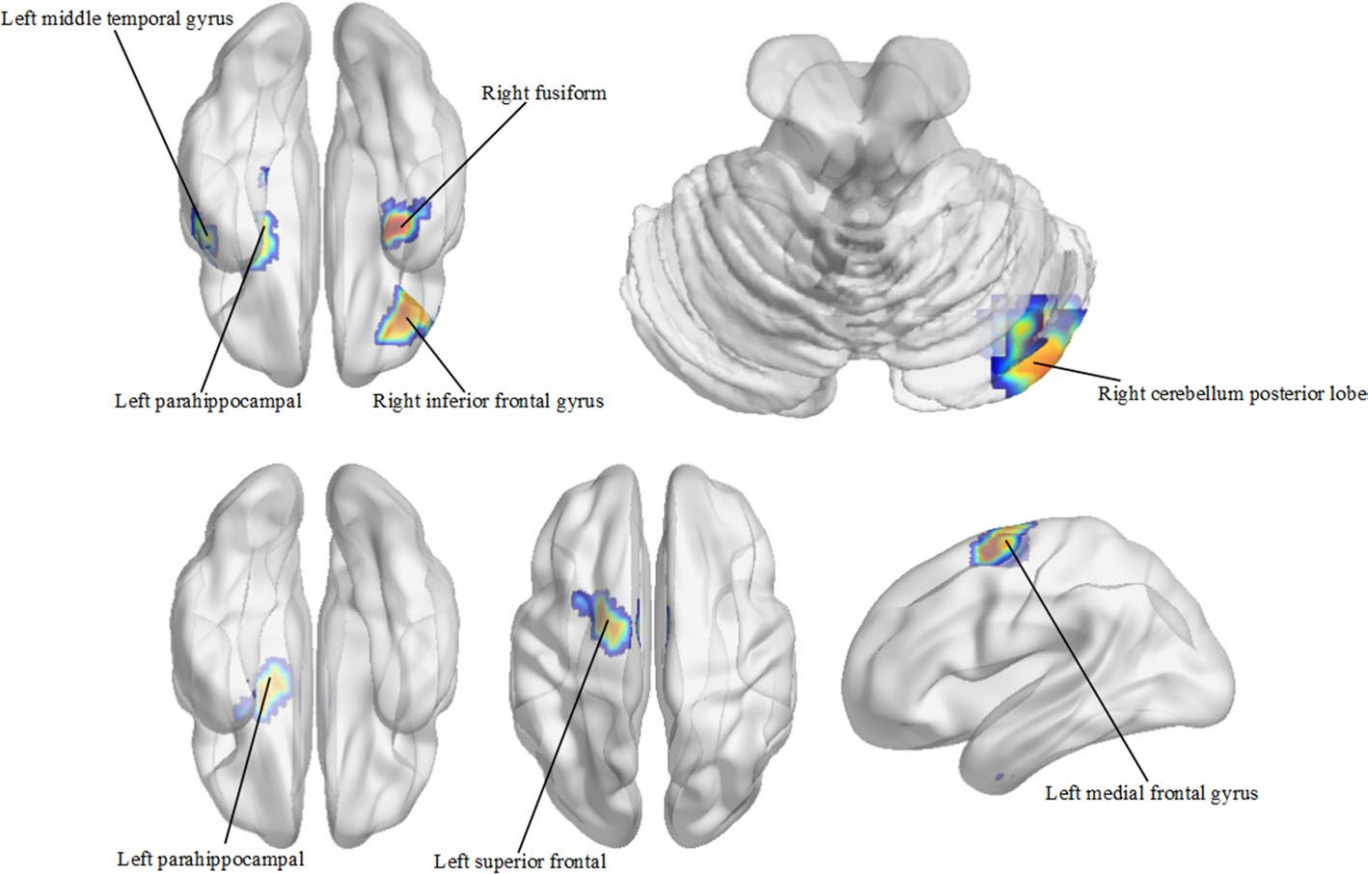
Clustering results (cluster size > 100) of remaining pixels (top 1%) in MRI (top) and PET (bottom) images

We obtained the features with the highest weight for sMRI and PET respectively through the above method. The highest weighted features for sMRI and PET were 145th and 133rd features, respectively. We input these features into the decoding network to obtain the key brain regions that make significant contributions to these features. Here, the higher the value of pixels is, the more important it is in the identification process. Thus, the value of pixels in the top 1% and cluster size > 100 remained (see Fig. 3). The most important brain regions were identified.

Results show that selected regions refer to the right inferior frontal gyrus, the right cerebellum posterior lobe, the left middle temporal gyrus, the right fusiform, and the left parahippocampal in sMRI [see Table 2 and Fig. 3 (top)]. As for PET, the selected regions include the left medial frontal gyrus, the left superior frontal, and the left parahippocampal [see Table 3 and Fig. 3 (bottom)].

**Table 3:** Cluster distribution statistics of PET 133rd feature deconvolution map in brain regions (3 clusters)

Region	MNI coordinates			Peak intensity	Voxels
	*x*	*y*	*z*		
Left parahippocampal	− 17	− 4	− 28	1.65	30
Left medial frontal gyrus	− 1	− 3	59	1.39	84
Left superior frontal	− 20	− 1	62	1.37	46

## Discussion

Recent technological advancements in data acquisition tools allowed neuroscientists to acquire data in a different modality. These data are huge in amount and complex in nature. It is an enormous challenge for data scientists to identify intrinsic characteristics of neurological big data and infer meaningful conclusions from these data. Mining such an enormous amount of data for pattern recognition requires sophisticated data-intensive machine learning techniques. Classical data mining techniques could be ineffective when problems get increasingly complicated. We propose a relatively lightweight model, which can efficiently extract meaningful features from medical images. It combines the characteristics of brain images, which can efficiently solve the correlation information between features, and fuse the information from different modal medical images. Our method increased accuracy, and the use of meaningful information.

### Comparison with previous methods

We compared our proposed method with previous AD diagnosis methods based on multimodal data, which used sMRI and PET images from the ADNI dataset as experimental data. To ensure a fair comparison, we reproduced their models using the parameters provided in their literature and conducted comparative experiments on the same dataset. As shown in Table 4, our proposed method outperforms the methods proposed by Feng et al. [17, 18], who used SBi-RNN and FSBi-LSTM to learn the correlation information between features, and Li et al. [23], who used a VGG-like network to mine multimodal image information. Our approach, which utilizes an improved Transformer to progressively learn global information of features, achieves superior performance in terms of accuracy.

**Table 4:** Comparison with previous research on AD diagnosis (mean ± standard deviation, %)

Methods	ACC	Precision	SPE	SEN	F1S	AUC
3DCNN + SBi-RNN [17]	93.33 ± 5.59	93.67 ± 7.22	94.91 ± 5.90	91.81 ± 8.01	92.52 ± 6.16	93.43 ± 5.56
3DCNN + FSBi-LSTM [18]	94.76 ± 5.70	95.78 ± 7.21	96.78 ± 5.53	93.76 ± 9.35	94.38 ± 6.12	94.85 ± 5.38
3D PMNet [23]	96.19 ± 4.92	98.89 ± 3.51	98.89 ± 2.78	93.19 ± 7.99	95.84 ± 5.40	96.47 ± 4.67
Proposed method	98.10 ± 2.46	99.09 ± 2.87	96.75 ± 5.28	95.82 ± 5.03	97.81 ± 2.87	98.35 ± 2.14

*ACC* accuracy, *PRE* precision, *SPE* specificity, *SEN* recall/sensitivity, *F1S* F1 score

### Visualization analysis

The brain consists of many brain regions responsible for different tasks. However, not all brain regions are related to AD. The most obvious pathological feature of AD is the loss of neurons, which is mainly manifested in the development of brain atrophy from AD signal area (such as the hippocampus and the temporal lobe) to the whole cortex [24]. Therefore, we try to find these brain regions by utilizing a different method from the traditional method of shielding brain ROIs, which may ignore some potential brain regions related to AD.

We made full use of the characteristics of the model to realize visualization and found the brain regions related to AD, which may help to better understand the potential pathogenesis of AD. Based on the visualization method in [20], we can obtain the weight of each feature through the attention matrix. According to the study of Zeiler et al., they realized the visualization of the model by deconvoluting the feature map output by convolution [25], and we can also do that on the final features.

Many studies suggest the temporal lobe transforms sensation into derived meaning to properly maintain visual memory, language understanding, and emotional association [26]. Brain atrophy in AD patients is symmetric and primarily affects medial temporal lobe structures [27]. Fusiform is part of the temporal lobe of the brain, this region is critical for face and body recognition. Convicted et al. [28] found that in AD, the volume of the temporal lobe is reduced, and the atrophy of the fusiform gyrus is the most obvious. Vidoni et al. [29] found that people with cognitive impairment had increased fusiform cortex engagement in visual coding tasks. Chang et al. [30] studied the relationships between regional amyloid burden and GM volume in AD and found pathological co-variance between the fusiform gyrus and para-hippocampus, and inferior temporal gyrus. Our structural findings are consistent with these previous studies and suggest that the etiology and mechanism of AD may be closely related to temporal lobe abnormalities.

The cerebellum plays an important role in motor function, controlling muscle tension and balance. It is a generally neglected area in the study of AD. However, there is increasing evidence that it is also involved in cognitive processing, emotion, and emotion regulation. The findings of Thomann et al. [31] confirmed that cognitive ability in AD patients was significantly associated with the volume of the posterior cerebellar lobe. Thus, we speculate that the aberrant cerebellar regions may be partially involved in the sluggishness and cognitive decline of AD.

The frontal lobe and hippocampus may be related to cognition and memory. According to the recent studies reported, the frontal lobe is responsible for logic, regulating behavior, complex planning, and learning. Alzheimer’s disease gradually damages the frontal lobe as the disease progresses. Laakso et al. [32] found that the volume of the hippocampus and left frontal lobe in the AD group was significantly smaller than that in CN subjects, and the decrease in left hippocampal volume was related to the decrease in MMSE score and the impairment of language memory. Our results for PET showed that the superior frontal gyrus, middle frontal gyrus, and parahippocampal may be subject to damage. Our results are consistent with previous studies. Especially, abnormal regions in the left hippocampus appeared in both sMRI and PET, which may suggest that the abnormality in this region is particularly related to AD. Our results might lead to an improved understanding of the underlying pathogenesis of the disease and provide valuable information for further research on AD.

## Conclusion

In this study, we proposed a framework based on 3DCNN and an improved Transformer for the diagnosis of Alzheimer's disease based on multimodal medical images. These promising results indicated that AD-related brain disorders can be precisely examined with multimodal medical images and deep learning techniques. We also strengthened the clinical interpretation of our proposed method through the visualization method, which may provide additional information to facilitate the diagnosis of AD.

## Methods

### Feature learning-based 3DCNN

The CNN has a powerful capability of local feature extraction. However, most CNN framework is designed for processing 2D images. For 3D brain images, they are usually processed into 2D slices, which will lose spatial information. To efficiently extract the abundant spatial information of 3D brain images, we adopt the 3D convolution kernel in this work. We alternatively stack the convolutional layers and pooling layers to get the multi-level features of multimodality brain images, as shown in Fig. 1.

In specific, the input image is convolved with a list of kernel filters in a convolutional layer. Then, a batch normalization layer is added between the activation function and convolution layer to improve the efficiency of training and avoid overfitting. We choose rectified linear unit (ReLU) as the activation function. Formally, we define the 3D convolution operation as follows:

1 $$ {\text{F}}_{j}^{l} (x,y,z) = {\text{Re}} {\text{LU}}(b_{j}^{l} + \sum\limits_{k} {\sum\limits_{{\delta_{x} }} {\sum\limits_{{\delta_{y} }} {\sum\limits_{{\delta_{y} }} {F_{k}^{l - 1} (x + \delta_{x} ,y + \delta_{y} ,z + \delta_{z} )} } } } *W_{kj}^{l} (\delta_{x} ,\delta_{y} ,\delta_{z} )), $$

where *x, y*, and *z* represent the voxel positions of a 3D image. ${W}_{kj}^{l}$ is the weight of the *j*th 3D kernel which connects the *k*th feature map of layer *l*1 with the *j*th feature map of layer l, ${F}_{k}^{l-1}$ is the *k*th feature map of the (*l*1)th layer, and ${b}_{j}^{l}$ is the bias term of the *j*th feature map of the *l*th layer. ReLU is employed as the activation function after the convolution of each layer. Finally, the output ${F}_{j}^{l}$ is obtained by summation of the response maps of different convolution kernels, which denotes the jth 3D feature map of the *l*th layer. To obtain more efficient and compact features, a max Max-pooling is used to down-sample the feature map after the convolution layer. Through the above operations, we can finally get a series of feature maps with rich 3D spatial information.

### Progressive learning of global feature information based on improved Transformer

Traditionally, the full connection (FC) layer is used to integrate the information of feature maps for the final identification. However, it just simply connects all neurons and cannot effectively take advantage of the spatial information from all feature maps.

Therefore, we choose to replace the FC layer with the encoder layer of the Transformer as in ViT [20]. However, unlike ViT, the input of the transformer module is not the image patches, but the feature maps extracted by 3DCNN. According to [33], the convolution operation itself has the ability to encode the position information. Therefore, we remove the position embedding mechanism replaced it with convolutional operations to perform positional encoding. Then, an encoder module of the Transformer is used to learn global correlation information between inputs.

The encoder block of the Transformer contains a multi-head self-attention (MSA) layer, and a feed-forward network (FFN) [19]. The normalization layer is applied before every block and residual connections are used after every block, as shown in Fig. 2.

#### Muti-head self-attention

The Self-attention (SA) mechanism is an important component of the transformer encoder block, which reduces the dependence on external information and is better at capturing the internal correlation of features [19]. This mechanism mainly solves the problem of long-distance dependence by calculating the interaction among embeddings. It allows each position in the sequence to attend to all other positions, enabling the model to consider the interdependencies between different elements. In simple terms, the self-attention mechanism calculates attention weights by computing the dot product between the query vector and the key vectors. Then, by scaling these weights and applying them to the value vectors, global correlation information between features is obtained. Finally, residual connections and feed-forward networks are used to enhance this information.

Unlike the classical SA module, we use convolutional operations instead of conventional linear mappings. Convolutional operations can preserve spatial information in the features and have fewer parameters than linear mappings, which can improve the computational efficiency of the model. Furthermore, 1 × 1×1 convolutions can also be used for positional encoding to help the Transformer differentiate the importance of different positions in the sequence during attention computation, as shown in Fig. 3.

Here, we use a convolutional kernel of size 1 × 1 × 1 to transform high-level features into Query(Q), Key(K), and Value(V) matrices in Fig. 3. Then, the Q, K, and V matrices are used to compute the attention weights, just like in the Transformer model. The calculation formula for a single-head self-attention is shown as follows:

2 $$ \begin{gathered} {\text{Attention}}(Q,K,V) = {\text{SoftMax}}\left( {\frac{{QK^{{\text{T}}} }}{\sqrt d }} \right){\text{V,}} \hfill \\ {\text{where Q = Conv}}_{1} (X),K{\text{ = Conv}}_{2} (X),\;V{\text{ = Conv}}_{3} (X). \hfill \\ \end{gathered} $$

Here, *X* represents the input features with a total of *N* samples, *d* represents the dimension of the features, and Conv represents a 3D convolution operation that maps the high-level features to *Q*, *K*, and V matrices using a 1 × 1 × 1 convolution kernel. In the calculation of attention weights, first, the inner product of the query and the key (*QK*^T^) are computed. Then, it is scaled by dividing it by the square root of the dimension of the query and key (√*d*). Finally, the SoftMax operation is applied to obtain the attention weights. The attention weights are then used to weight the values, and their weighted sum yields the final output. A single-head SA layer has limited capability to focus on a specific entity (or several entities). Thus, several self-attention heads are used in MSA layers to allow the learning of different kinds of interdependencies. The calculation formula for multi-head self-attention (MSA) module is shown as follows:

3 $$ \begin{gathered} {\text{MultiHead}}(X) = {\text{Concat}}({\text{head}}_{1} ,{\text{head}}_{2} ,...,{\text{head}}_{{_{h} }} ) \hfill \\ {\text{where head}}_{i} = {\text{Attention}}({\text{Conv}}_{1}^{i} (X),{\text{Conv}}_{2}^{i} (X),{\text{Conv}}_{3}^{i} (X)) \hfill \\ \end{gathered} $$

After MSA, there is a residual connection to preserve the original information of the input features. Here, we represent the features after residual connection as

4 $$ X{\prime} = \alpha O + X, $$

where *O* is the output feature, *X* the input feature, and *α* a learnable scalar. Initially, we set the value of *α* to 0, so that the self-attention mechanism is masked at the beginning of model training, allowing the 3DCNN to focus on local feature extraction. As training progresses, the value of *α* increases, and the model starts to learn global correlation information between features. The maximum value of *α* is 1.

#### Feed-forward networks

After each layer passes through attention, there will be an FFN, which is used for spatial transformation. The FFN contains two linear transformation layers with the ReLU activation function. The FNN performs dimension expansion/reduction and nonlinear transformation on each token to enhance the representation ability of the tokens, thus increasing the performance ability of the model:

5 $$ {\text{FFX(X) = max(0,}}XW_{1} { + }b_{1} {)}W_{2} { + }b_{2} , $$

where  is the weight of the first layer, which projects *X* into a higher dimension *D*.  is the weight of the second layer, and *b*_1_ and *b*_2_ are the biases.

The output of the transformer layer is transformed linearly through the MLP layer, and finally identified by the SoftMax function.

### Network model framework

**Fig. 4:**
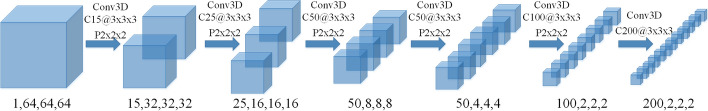
The architecture of deep 3D CNNs denoted with the sizes of each layer’s input, convolution, max pooling, and output layers and the numbers and sizes of generated feature maps. C is a convolutional layer, the P is max pooling layer, @ is the number of filters such as 15@ 3 × 3 × 3 is 15 filters whose size are 3 × 3 × 3 and P 2 × 2 × 2 is pooling layers, with a size of 2 × 2 × 2. The number below each layer represents the shape of the feature

In this experiment, we use 3DCNN to extract features of sMRI and PET. To extract the differential information of sMRI and PET, we built and trained a 3DCNN network for sMRI and PET respectively, while they share the same network structure. We obtained 200 features with dimensions of 2 × 2 × 2 after applying 3DCNN. Each feature represents one part of the brain. Then, the encoder block of the Transformer is used to extract interactive information among various features instead of the traditional FC layer. Here, sMRI and PET feature also shared the same transformer module. Finally, the learned information was concatenated and further passed to MLP for disease diagnosis. The overall framework of the network model is shown in Fig. 4. The 3DCNN and the Transformer framework were simultaneously trained in the end-to-end framework.

### Model visualization

Given the complexity and high risk of medical decision-making, model interpretation is particularly important for medical imaging applications. Incorrect diagnosis or failure to detect diseases could be detrimental to patients, and therefore, it is necessary to explain the reasons behind the decisions made by deep learning models.

**Fig. 5:**
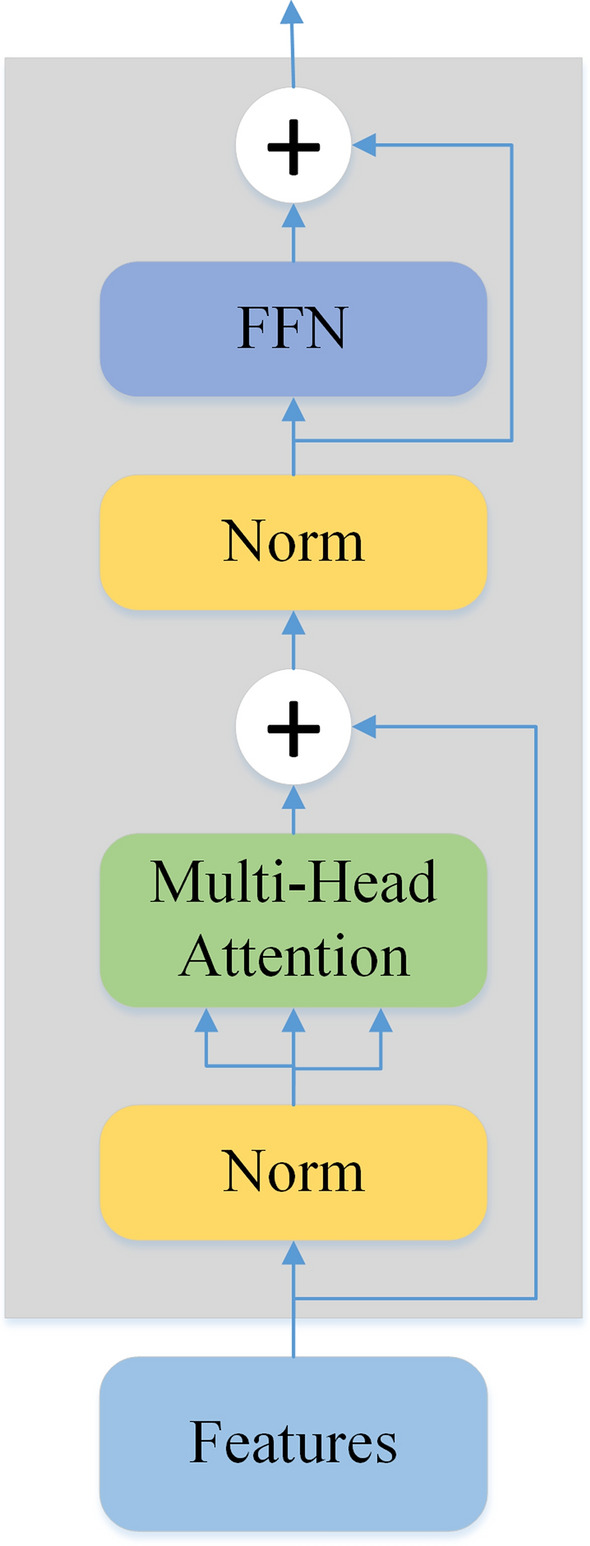
The struct of transformer encoder

It has been verified that through the relevance of a feature to identification, the identifiable power of the feature can be quantitatively measured by the attention matrix. Then, the important brain region can be obtained by decoding the feature through a deconvolution network [25]. The process of visualization is shown in Fig. 5.

#### Get the feature with the highest weight

The Attention Rollout is used to compute the attention map from output tokens to input features. In specific, since the residual connections in the self-attention and FFN layers of the Transformer modules (as shown in Fig. 2) play an important role in connecting the corresponding positions across different layers, we add extra weights to represent the residual connections for computing the attention rollout map as follows:

6 $$ A \, = \, 0.5W + \, 0.5I. $$

In the formula, *A* represents the attention matrix considering the residual connections, *W* represents the original attention matrix, and *I* represent the identity matrix. Considering that the residual connection parameter *α* after the MSA layer approaches 1 (0.996) in the later stages of training, the weight of the residual is set to 0.5. Finally, to calculate the attention from the feature input layer to the output layer, we recursively multiply the attention matrices of previous layers in all subsequent layers. The formula for calculating the attention rollout for the ith layer is shown below.

7 $$ \tilde{A}(l_{i} ) = \left\{ {\begin{array}{*{20}ll} {A(l_{i} )\tilde{A}(l_{i - 1} )} & {if \, i \, > j} \\ {A(l_{i} )} & {if \, i \, = j} \\ \end{array} } \right. $$

$\tilde{A}(l_{i} )$ represents the attention calculation of the *i*th layer during the attention rollout process, which is multiplied with the layer attention matrix A through matrix multiplication. Each row of the matrix represents the attention weight between a feature and other features. Then, the average of all attention matrices is taken along the row and column dimensions, resulting in a 200-dimensional vector. This vector indicates the contribution weights of the 200 different features of the same modality to the classification results. The feature with the highest weight is then selected for subsequent visualization research.

#### Feature decoding

To find out the relevant brain regions for AD diagnosis, two deconvolution networks (for sMRI and PET) are trained whose structures were mirror images of the 3DCNN parts. Deconvolution networks can restore the features extracted by 3DCNN to the original image. Thus, we transform the features with the highest weight into pixels using the trained deconvolution networks for analysis.

### Data and preprocessing

In this experiment, we used the open-access sMRI and PET datasets from Alzheimer’s Disease Neuroimaging Initiative (ADNI) database^1^. ADNI is multicenter research to search for clinical, imaging, genetic, and biochemical biomarkers for the discovery of AD. We used the 18F-FDG-PET and sMRI data downloaded from ADNI with each pair of FDG-PET and sMRI for the same subject captured at the same time. All sMRI scans (T1-weighted MP-RAGE sequence at 1.5 T) used in our work were acquired from 1.5 T scanners and typically consisted of 256 × 256 × 176 voxels with a size of approximately 1 mm × 1 mm × 1.2 mm. The PET images have many different specifications, but they were finally processed into a unified format.

Our dataset consists of 210 subjects, consisting of 88 AD subjects and 122 cognitively normal (CN) subjects. The male to female ratio is 106/104. The age of the subjects ranges from 56 to 92, and there is no difference in age and gender between AD and CN subjects (*p* = 0.0513). Some previous studies did not consider the balance of gender and age, so the features extracted from the data may not be related to disease, which may be related to gender or age, so we strictly controlled for their balance. The characteristics of the subjects are summarized in Table 5.

**Table 5:** Characteristics of the subjects in the ADNI dataset (mean ± standard deviation)

	AD	CN
Gender (M/F)	46/42	60/62
Age (years)	75.43 ± 8.20	77.42 ± 6.48
MMSE	22.32 ± 2.67	28.93 ± 1.32
Global CDR	0.86 ± 0.31	0.10 ± 0.20

For the sMRI data, we conducted Anterior Commissure (AC) – Posterior Commissure (PC) reorientation via MIPAV software.^2^ Tissue intensities inhomogeneity is then corrected using the N3 algorithm [34]. Skull stripping, cerebellum removal, and three main tissues [gray matter (GM), white matter (WM), and cerebrospinal fluid (CSF)] segmentation were conducted via the Cat12 tool of SPM12^3^. Existing research shows that GM demonstrated higher relatedness to AD [35, 36]. Therefore, we chose the GM masks in this work. Finally, we used the hierarchical attribute matching mechanism for the elastic registration (HAMMER) algorithm [37] to spatially register the GM masks to the template of the Montreal Neurological Institute (MNI) 152 [38].

For the PET, first, we realigned them to the mean image. Then, we registered it to the corresponding sMRI image. Finally, in common with sMRI images, they were registered to the MNI152 brain atlas.

Finally, all the sMRI and PET data were smoothed to a common resolution of 8-mm full-width at half-minimum. And they were all down-sampled to 64 × 64 × 64.

## Experiment settings

The ranges of pixel values of each sMRI or PET are different, hence, we normalized the preprocessed sMRI and PET images to the same range for a subject. We used min–max normalization to scale all pixel values into 0–1 as follows:

8 $$ z = \frac{x - \min (x)}{{\max (x) - \min (x)}}, $$

where *z* is the normalized pixel values for sMRI or PET.

As shown in Fig. 1, the 3DCNN part consisted of 5 stacked convolutional and max-pooling layers. A separate convolution layer was used as the last layer. A batch normalization layer and an ReLU activation function were added after each convolution layer. We set all convolutional layer strides to 2 and padding was set to be the same as layer input. The structure of 3DCNN for sMRI and PET The structure of 3DCNN used to extract MRI and pet features is the same, but they do not share parameters. In the Transformer part, we chose the encoder block of the framework, but the number of heads is set at 4. To avoid overfitting, we just stacked two layers of the transformer encoder block. Then two linear transformations were performed in the MLP part, and a dropout with probability of 0.1 was performed after each linear transformation. We chose Adam optimizer (with default parameters) to optimize the model parameters and categorical cross-entropy as the loss function, which is suitable for identification tasks. We set the batch size to 11, the number of epochs to 60, and the learning rate to 10^−4^. The learning rate was decaying every 20 epochs, and the decay factor was set to 0.1. We set the random number seed for experiment debugging.

A tenfold cross-validation algorithm was adopted to evaluate the identification performance. In specific, all samples were randomly divided into 10 portions to evenly distributed AD and CN data in every portion. Then, samples from two portions were used as the testing data (21 subjects) and the validation data (21 subjects) respectively, while the rest were utilized as the training data (168 subjects). The cross-validation algorithm was applied and the final identification accuracy was obtained by averaging the results of 10 tests.

**Fig. 6:**
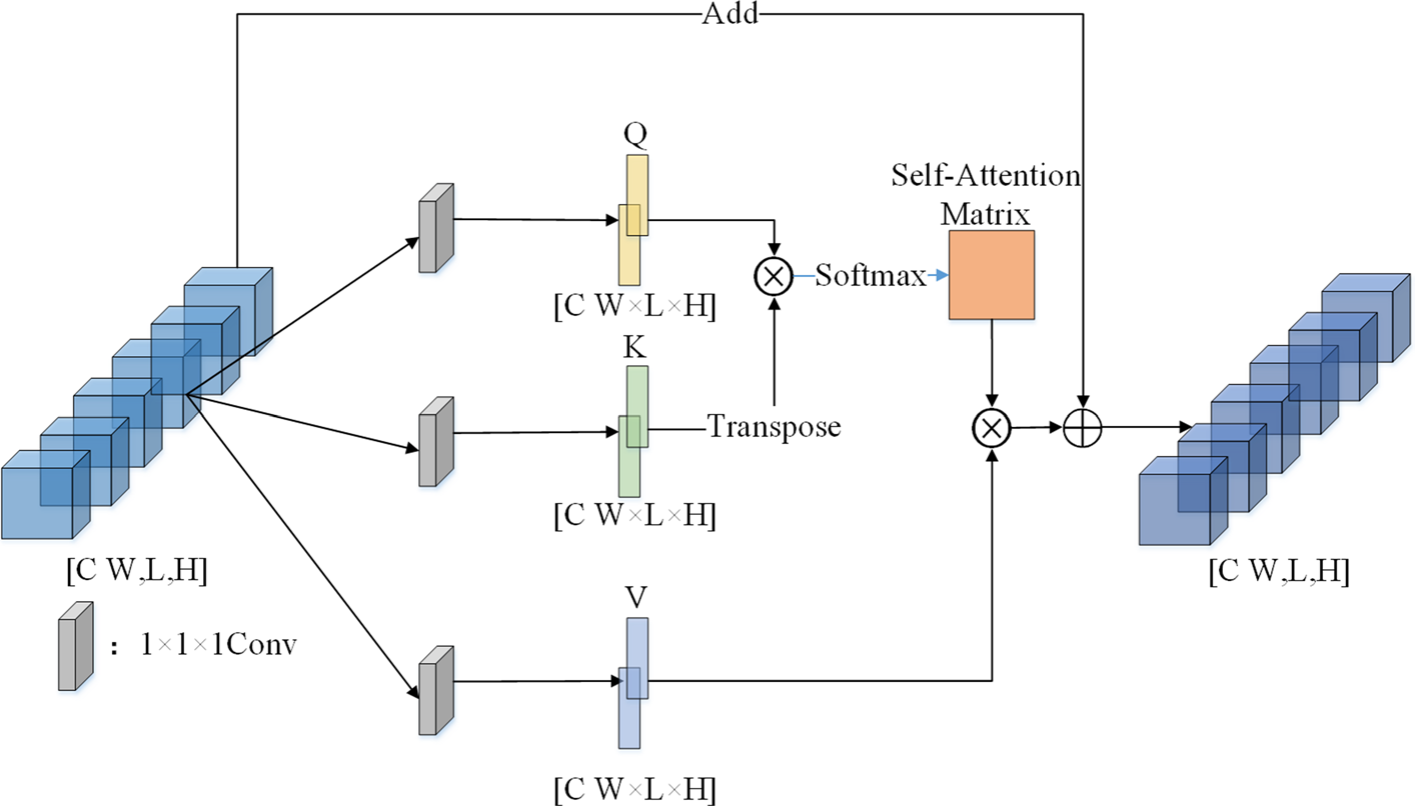
Convolution-based self-attention mechanism

**Fig. 7:**
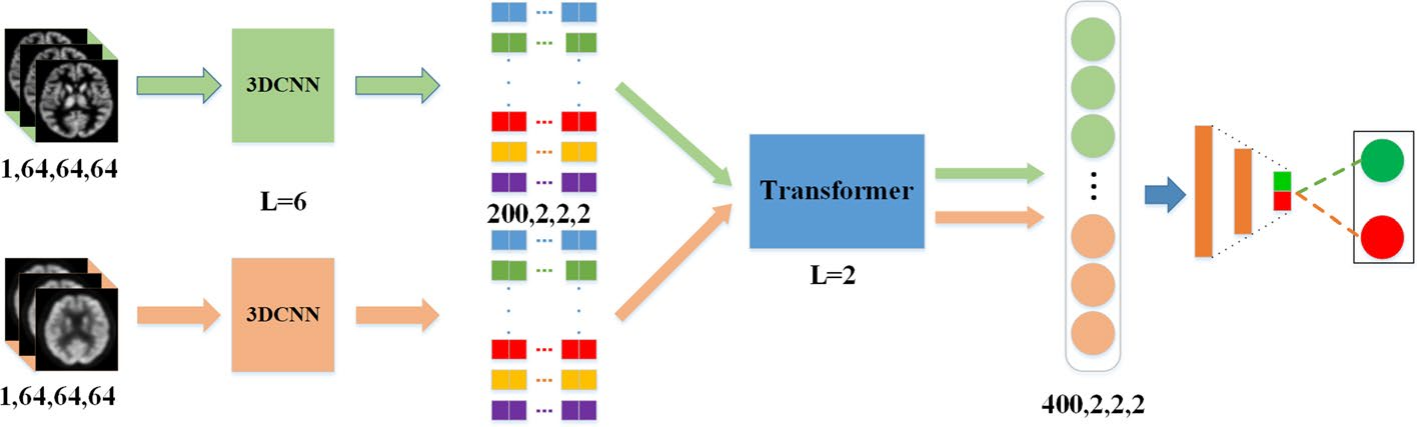
The framework of network model

**Fig. 8:**
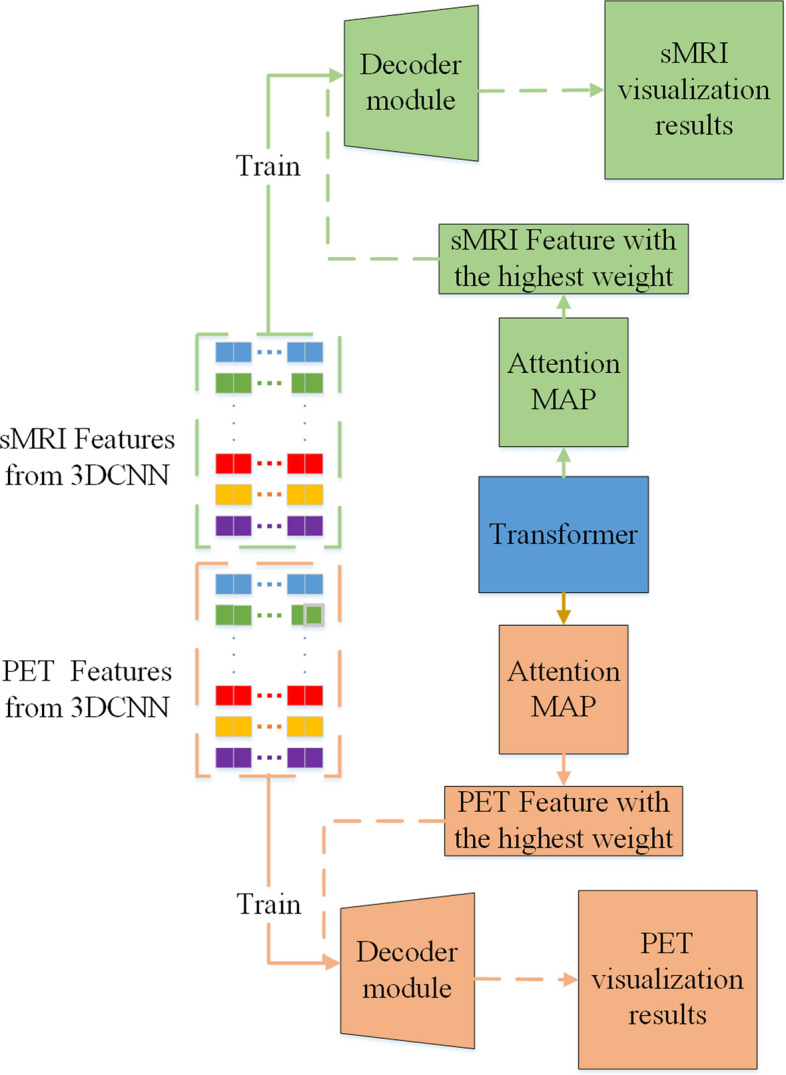
Visualization framework

In the cross-validation scheme, the model parameters and features were not necessarily the same across all loops. Several parameters were used to evaluate the identification performance, including accuracy (ACC), precision (PRE), specificity (SPE), recall/sensitivity (SEN), F1 score (F1S), and area under receive operation curve (AUC). PRE indicated how many of the positive values predicted by the model are positive. F1 is the harmonic average of accuracy and recall/sensitivity, which was a comprehensive evaluation index. AUC can intuitively evaluate the quality of the identifier (Figs. 6, 7, 8).

For deconvolution networks in visualization, we set the batch size to 20, the number of epochs to 3000, and the learning rate to 10^−4^. The learning rate was decay every 500 epochs with the decay factor of 0.5. Adam optimizer was used to speed up training. We utilized the nearest neighbor interpolation algorithm as the up-sampling algorithm and the mean square error (MSE) loss as the loss function, which could better measure the reconstruction error.

## Data Availability

Data collection and sharing for this project were funded by the Alzheimer’s Disease Neuroimaging Initiative (ADNI). This investigation was led by Michael W. Weiner (Michael.Weiner@ucsf.edu) and the complete list of collaborators can be found at https://adni.loni.usc.edu/wp-content/uploads/how_to_apply/ADNI_Acknowledgement_List.pdf. ADNI is funded by the National Institute on Aging, the National Institute of Biomedical Imaging and Bioengineering, and through generous contributions from the following: AbbVie, Alzheimer’s Association; Alzheimer’s Drug Discovery Foundation; Araclon Biotech; BioClinica, Inc.; Biogen; Bristol-Myers Squibb Company; CereSpir, Inc.; Cogstate; Eisai Inc.; Elan Pharmaceuticals, Inc.; Eli Lilly and Company; EuroImmun; F. Hoffmann-La Roche Ltd and its affiliated company Genentech, Inc.; Fujirebio; GE Healthcare; IXICO Ltd.; Janssen Alzheimer Immunotherapy Research & Development, LLC.; Johnson & Johnson Pharmaceutical Research & Development LLC.; Lumosity; Lundbeck; Merck & Co., Inc.; Meso Scale Diagnostics, LLC.; NeuroRx Research; Neurotrack Technologies; Novartis Pharmaceuticals Corporation; Pfizer Inc.; Piramal Imaging; Servier; Takeda Pharmaceutical Company; and Transition Therapeutics. The Canadian Institutes of Health Research is providing funds to support ADNI clinical sites in Canada. Private sector contributions are facilitated by the Foundation for the National Institutes of Health (www.fnih.org). The grantee organization is the Northern California Institute for Research and Education, and the study is coordinated by the Alzheimer’s Therapeutic Research Institute at the University of Southern California. ADNI data are disseminated by the Laboratory for Neuro Imaging at the University of Southern California. The dataset of this paper was obtained from Alzheimer’s Disease Neuroimaging Initiative (ADNI^4^).

## Abbreviations

AD: Alzheimer’s disease
sMRI: Structural magnetic resonance imaging
PET: Positron emission tomography
3DCNN: Three-dimensional convolutional neural network
ADNI: Alzheimer’s disease neuroimaging initiative
SBi-RNN: Stacked bidirectional recurrent neural network
FSBi-LSTM: Stacked bidirectional long short-term memory
ViT: Vision Transformer
ReLU: Rectified linear unit
FC: Full connection
MSA: Multi-head self-attention
FFN: Feed-forward network
CN: Cognitively normal
AC: Anterior commissure
PC: Posterior commissure
GM: Gray matter
WM: White matter
CSF: Cerebrospinal fluid
MNI: Montreal neurological institute
ACC: Accuracy
PRE: Precision
SPE: Specificity
SEN: Sensitivity
F1S: F1 score
AUC: Area under receive operation curve
MSE: Mean square error

## Footnotes

1 https://adni.loni.usc.edu.

2 https://mipav.cit.nih.gov.

3
www.fil.ion.ucl.ac.uk/spm/.
